# Interaction of Proteins with Inverted Repeats and Cruciform Structures in Nucleic Acids

**DOI:** 10.3390/ijms23116171

**Published:** 2022-05-31

**Authors:** Richard P. Bowater, Natália Bohálová, Václav Brázda

**Affiliations:** 1 School of Biological Sciences, University of East Anglia, Norwich Research Park, Norwich NR4 7TJ, UK; r.bowater@uea.ac.uk; 2 Department of Biophysical Chemistry and Molecular Oncology, Institute of Biophysics of the Czech Academy of Sciences, 61265 Brno, Czech Republic; nataliabohalova@gmail.com; 3 Department of Experimental Biology, Faculty of Science, Masaryk University, Kamenice 5, 62500 Brno, Czech Republic

**Keywords:** cruciform, DNA base sequence, DNA structure, DNA supercoiling, epigenetics, genome stability, inverted repeat, replication, transcription

## Abstract

Cruciforms occur when inverted repeat sequences in double-stranded DNA adopt intra-strand hairpins on opposing strands. Biophysical and molecular studies of these structures confirm their characterization as four-way junctions and have demonstrated that several factors influence their stability, including overall chromatin structure and DNA supercoiling. Here, we review our understanding of processes that influence the formation and stability of cruciforms in genomes, covering the range of sequences shown to have biological significance. It is challenging to accurately sequence repetitive DNA sequences, but recent advances in sequencing methods have deepened understanding about the amounts of inverted repeats in genomes from all forms of life. We highlight that, in the majority of genomes, inverted repeats are present in higher numbers than is expected from a random occurrence. It is, therefore, becoming clear that inverted repeats play important roles in regulating many aspects of DNA metabolism, including replication, gene expression, and recombination. Cruciforms are targets for many architectural and regulatory proteins, including topoisomerases, p53, Rif1, and others. Notably, some of these proteins can induce the formation of cruciform structures when they bind to DNA. Inverted repeat sequences also influence the evolution of genomes, and growing evidence highlights their significance in several human diseases, suggesting that the inverted repeat sequences and/or DNA cruciforms could be useful therapeutic targets in some cases.

## 1. Introduction

**Figure 1:**
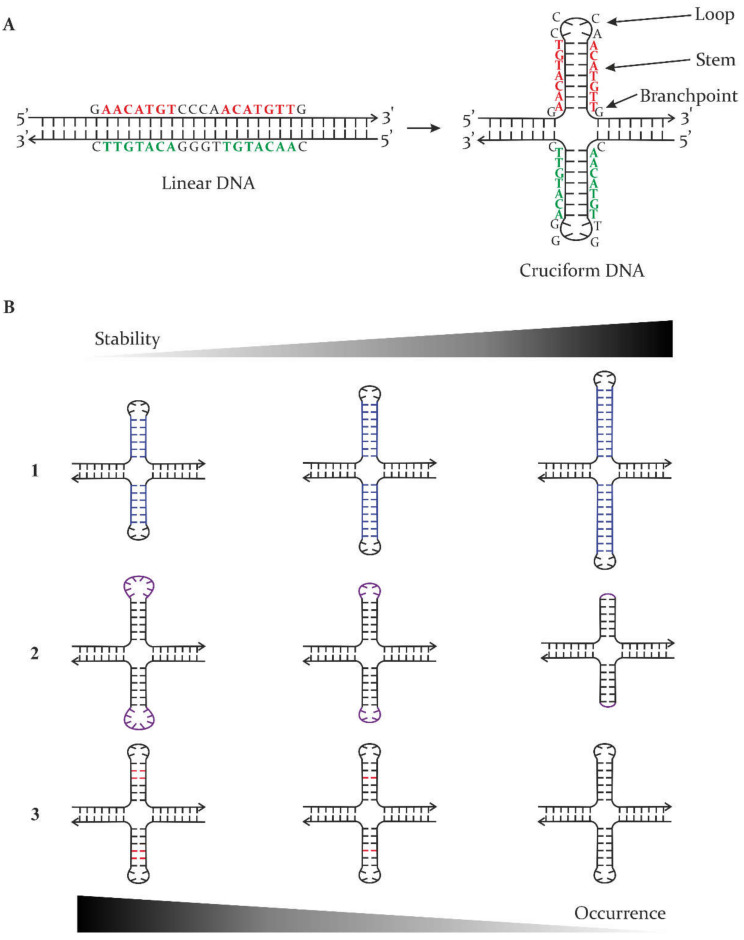
Inverted repeat sequences can form different types of double-stranded conformations. (**A**) Transition of inverted repeat in a linear conformation to a double hairpin, cruciform state. For the sequence indicated, the cruciform structure consists of four branchpoints and two 7 bp-long stems, each with 4 nt loops. (**B**) Decisive factors for the resulting thermodynamic stability and genomic occurrence of cruciform structures are: (1) stem size indicated in blue; (2) loop length indicated in purple; (3) possible mismatches in base pairing indicated in red. The arrows at the top and bottom of part (B) highlight the relative stability and occurrence of the represented cruciforms, with the larger and darker part of the arrows indicating those that are most stable and are most likely to occur in genomes. For all schematic molecules, the arrow indicates the 3′-end of the DNA strand.

The wealth of DNA sequence information provided by genome sequencing projects has brought new insights into the primary sequences of genomes and also about possible sequence-dependent local secondary structures [1]. The primary base sequence alone is insufficient to decipher all principles that support basic molecular processes and those that maintain genomic and cellular stability. Inevitably, in-depth knowledge of epigenetic modifications and the local and global DNA structure is crucial for a full understanding of these processes. DNA molecules typically form two-stranded, right-handed helical B-form structures, which maximize the thermodynamic stability of the molecule [2]. However, a range of alternative (non-B) structures can also occur in DNA, and these are usually characterized by the occurrence of single-stranded regions (loops) and/or sites of disrupted base pair stacking (junctions between continuous B-form DNA and the alternative structure) [3]. Any disruption of stacking interactions or hydrogen bonds in base pairs alters the thermodynamic stability of the molecule, but non-B DNA structures can be favourable for some sequences under some environmental (and cellular) conditions. Although they were initially considered as in vitro artefacts, several local secondary DNA structures are now well characterized and confirmed to form in living cells under physiologically relevant conditions [4,5,6]. These sequence-dependent conformational changes give rise to triplexes [7,8], G-quadruplexes [5,9], i-motifs [10], R-loops [8,11], four-way junctions [12], and cruciforms [13,14,15]. The latter is formed in DNA molecules containing inverted repeat sequences, either uninterrupted or interspaced with several additional bases forming loops. Thus, cruciform structures consist of branch-points, stems, and loops (Figure 1A) [15]. The thermodynamic stability of cruciforms is influenced by their size, with stable cruciforms usually requiring the inverted repeat to be at least six bases in length (for the stem, or one half of the repeat). Cruciforms can also arise from imperfect inverted repeats, meaning that unpaired bases occur within the stems of the cruciform, although this means such structures are energetically disfavoured compared to the fully base-paired structure [15,16]. In addition to inverted repeat unit size and unpaired bases, the length of the loop is also a critical factor influencing the stability of such structures (Figure 1B). Analyses of inverted repeats in various genomes have shown they have a non-random distribution and a functional association with regulatory sites, including promoters [17,18].

Inverted repeats and cruciforms have been found in all forms of life and appear to share similar functions and properties in many of them [3,6,17,18,19,20,21]. Inverted repeats are found in bacteria, eukaryotes, archaea, and viruses in higher amounts than would be expected from a random distribution of bases in both coding and non-coding regions, with a more pronounced frequency in non-coding regions. The frequency of inverted repeats in all organisms decreases with increasing length, but in most cases, the relative difference between expected and actual numbers tends to be higher for longer repeats [18]. As we describe in detail below, in all organisms, the presence of inverted repeats contributes to reduced genomic stability, primarily through the induction of inversions and the formation of hairpins and four-way junctions, which induce the stalling of polymerases and the generation of double-strand breaks. Cruciform conservation across all domains is, thus, a likely result of their involvement in essential molecular processes, such as opening of the DNA double helix during replication, transcription, and DNA damage repair [15].

## 2. Biophysical and Molecular Characterization of Cruciforms

The formation of cruciforms during the expression of genes was first postulated more than 50 years ago [22]. Their presence and function was subsequently studied both in vitro and in vivo, mostly for those located in plasmid DNAs from bacteria and yeasts [15]. The formation of cruciform structures requires the double-stranded helix of DNA to be opened, an energetically unfavourable process. A wide range of chemical and molecular probes have characterized properties that influence this process [6,23], with computer modelling methods helping to interpret experimental data [24]. Biophysical and molecular studies have clearly demonstrated that cruciforms are stable for some DNA molecules in vitro, but the situation has been less clear in vivo, mainly due to difficulties with studying the DNA structure inside cells. To assay for cruciform structures in cells, a range of probes of DNA structure have been used, including various factors that attack single-stranded regions of DNA, including psoralen and UV light cross-linking [6,25,26]. In some cases, the experiments cause the death of the cells, leading to studies being referred to as in situ to highlight that the cells are under physiological conditions, but may no longer be “living” [27]. Using *Escherichia coli* as a model, experiments have shown that large inverted repeats can be detected in cruciforms under some conditions, but sometimes at relatively low proportions of the total DNA [6]. Direct visualization of cruciforms in cells was attempted with a monoclonal antibody (2D3) shown to recognize cruciforms, but not heteroduplex slipped-stranded DNA containing a hairpin on one strand only [6,27]. Immunoprecipitation using this antibody revealed the presence of cruciform-containing DNA at a yeast replication origin, although it is unclear whether it specifically binds cruciforms or a panel of slipped-stranded DNA molecules [6,28]. Many methods continue to be used to study cruciform structures and their formation, from broad bioinformatic studies and electrophoretic in vitro assays to in vivo visualization by specific antibody interaction and single-molecule-level analyses [29,30,31]. Indeed, single-molecule manipulation of DNAs allowed cruciform formation, dynamics, and removal to be studied in real-time [32,33], as well as to reveal the mechanochemical properties of cruciform structure and cooperativity between opposing stem–loop structures [34].

**Figure 2:**
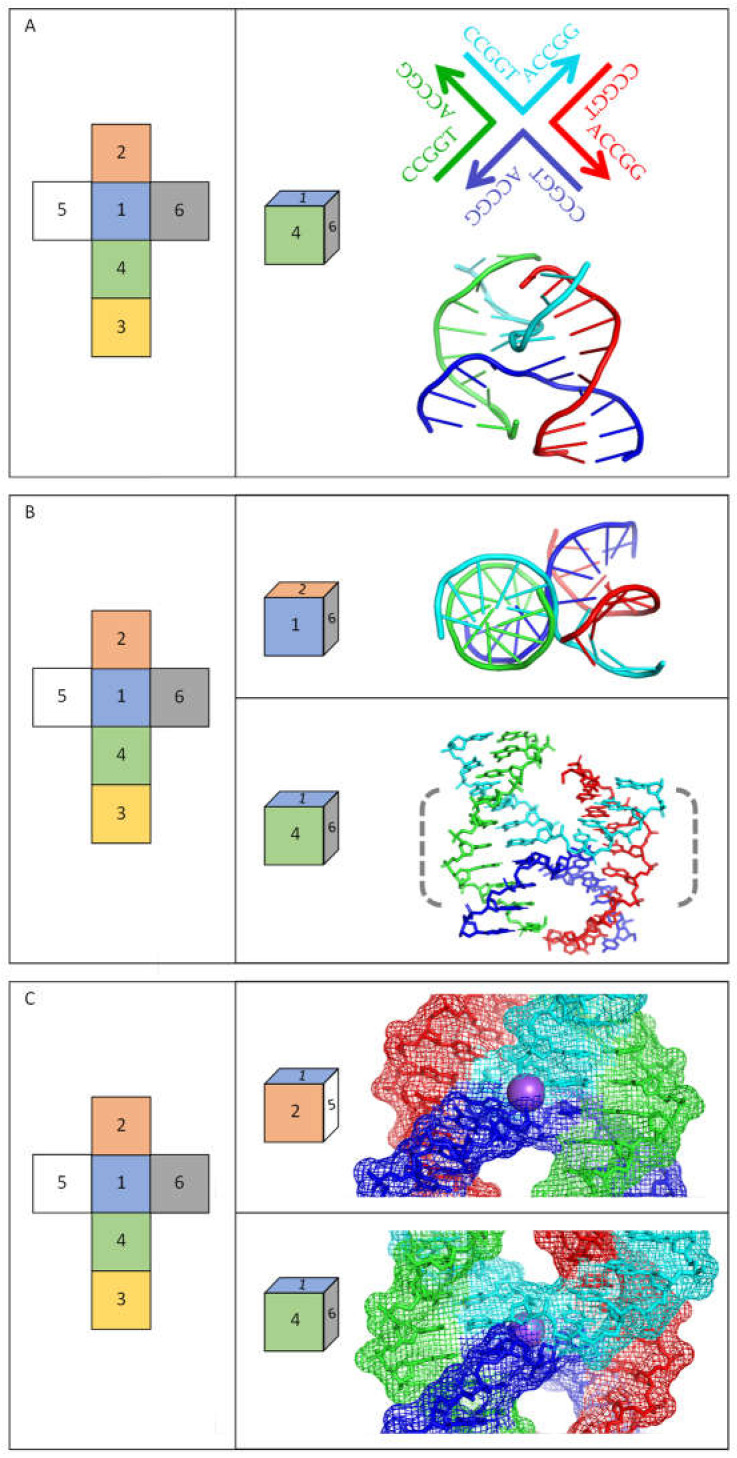
High-resolution structure of a cruciform (four-way junction) formed by an inverted repeat DNA sequence. Images show the X-ray crystallographic structure determined at 2.10 Å for DNA with the sequence 5′-CCGGTACCGG-3′ (1DCW) [37]. The DNA alone forms a four-way junction in a stacked-X conformation, in which duplexes coaxially stack, with each pair of stacked duplexes related by +30° to +60° (right-handed) rotation. The continuous (least distorted relative to B-DNA) strands are coloured as green and red, while those of the crossing strands (making a tight U-turn) are coloured blue and cyan. In each panel, the images show the structure visualised via different axes viewpoints as indicated by the coloured squares. (**A**) The upper image provides a schematic view of the molecule, the distinct strands (in different colours), and their sequences, with arrows indicating the 3′-ends of the DNA strands. The lower image presents the high-resolution structure of 1DCW, illustrating its arrangement of base pairs. (**B**) The upper image views the structure down the helix axis of one pair of stacked duplexes, while the lower image views it from a rotational shift of approximately 90°. (**C**) The images zoom in on the central part of the structure (dashed bracketed region in (**B**)) to highlight the electrostatic interactions, particularly close to the Na^+^ ion at its centre. The lower image views the same face of the dyad axis shown in (**B**), and the upper image shows the opposite face of the axis, viewed from a rotational shift of approximately 180°.

In recent years, advances have been especially striking in high-resolution analyses of non-B DNA structures either as the nucleic acid alone or in combination with proteins. In the context of this review, significant progress has been made in studies of four-way junctions, which are equivalent to the central part of cruciform structures—see Figure 1. Four-way junctions (often referred to as Holliday junctions) are critical intermediates in many DNA recombination and repair pathways [35], but it is important to recognize that such structures are usually formed by DNA molecules that do not contain inverted repeat sequences. A range of structural studies demonstrate that four-way junctions adopt different structures depending on the ionic environment and other factors [35,36]. X-ray crystallography and nuclear magnetic resonance (NMR) analyses of several DNA inverted repeat sequences confirm that they adopt the “stacked-X structure” in the absence of proteins, in which duplexes coaxially stack on each other. In thermodynamic terms, this type of structure has the most favourable energetics when monovalent or divalent cations are available to counteract the repelling interactions that occur between the negatively charged backbones, although cations are not an absolute requirement for the formation of stable cruciform structures. Figure 2 shows several views of a DNA inverted repeat structure determined at 2.10 Å for the sequence 5′-CCGGTACCGG-3′ [37], and similar structures have been observed for a variety of other inverted repeats [36]. The DNA forms a four-way junction in a “stacked-X” conformation (Figure 2). Two strands are “continuous” and are closest to a B-DNA conformation, while the other two strands make a tight U-turn and cross at the junction. The stacked-X structure is seen clearly in Figure 2A,B. For this complex, a Na^+^ ion at its centre reduces electrostatic repulsion as the phosphodiester backbones come close to each other at the junction crossover (Figure 2C). Note that when the stacked-X structure is viewed from one face, Na^+^ is relatively protected by the DNA backbones, but it is relatively accessible to the local environment from the opposite side. Molecular dynamics simulation of a decamer inverted repeat as a four-way junction confirms its twofold symmetry and that temperature and its structural integrity are preserved by a range of other parameters (i.e., the presence of ions, solvents, etc.) [38]. Epigenetic markers on DNA, such as hydroxymethyl and methyl substituents, can be accommodated without disrupting the structure or stability of the cruciform, although they open the structure to make the junction core more accessible [36]. The binding of proteins—usually enzymes—to four-way junctions can alter their conformation, although they can have dramatically different effects [36,39,40,41]. High-resolution structures that are currently available for these altered conformations of four-way DNA junctions with proteins bound are usually for sequences that are not inverted repeats. It is expected that DNA cruciforms formed by inverted repeats will have similar flexibility when proteins bind to them, but this still has to be verified by high-resolution structures.

## 3. Presence of Inverted Repeats in Genomes

The various experimental methods referred to above have provided abundant evidence for the presence of inverted repeats in genomes across all forms of life [6,42]. Since the start of the 21st Century, the evidence has improved due to dramatic advances in sequencing technologies and bioinformatic analyses identifying genome sequences for many different organisms. Notably, it has been challenging to accurately sequence genomic regions that are rich in repeated bases for various reasons, but potentially including the presence of thermodynamically stable secondary structures [43]. Recent advances in sequencing methods mean that such problems can now usually be resolved, even for the human genome [44,45]. Here, we summarize the deepening understanding about the amounts of inverted repeats across all forms of life.

### 3.1. Viruses

**Figure 3:**
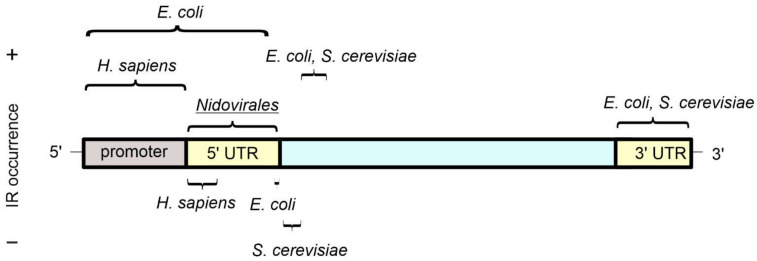
The occurrence of inverted repeat sequences in gene features as determined by bioinformatic analyses. An idealised gene and its regulatory sequences are shown, with UTR referring to “untranslated regions”. A relative abundance (+) or depletion (−) of inverted repeats in the indicated genomes is highlighted above and below the idealised gene, respectively. For *E. coli* and *S. cerevisiae*, inverted repeats with a stem length from 5 bp and a spacer length up to 8 bp were considered [50,51], while for *H. sapiens* and viruses from the *Nidovirales* order, inverted repeats with the stem length from 6–30 bp and spacer length up to 10 bp were taken into account [18,42].

Inverted repeats are found in higher numbers in many viral genomes than is expected from a random occurrence of bases [46]. This is true for many different types of viruses, but we illustrate this using Severe Acute Respiratory Syndrome Coronavirus 2 (SARS-CoV-2) and adeno-associated viruses (AAV), which have single-stranded RNA and DNA genomes, respectively. Using the SARS-CoV-2 virus genome as an example, a total of 1203 inverted repeats with stems of 6-13 bp in length were identified. The average frequency of their occurrence was 40.24 inverted repeats per 1000 nt, whereas it was 33.90 for the entire *Nidovirales* family to which SARS-CoV-2 belongs [42]. Recurrent mutations were shown to occur within inverted repeats with a higher frequency than would be expected from a random distribution of them [47,48]. Furthermore, an abundance of inverted repeats was found within 5′ untranslated regions of the *Nidovirales* family (Figure 3) [42]. In a different virus, AAV, terminal inverted repeats of 125 bases can form T-shaped hairpin structures by base-paring of two small internal inverted repeat sequences and large flanking inverted repeat sequences [49]. This terminal inverted repeat of AAV was determined as the binding site for several transcriptional transactivators and was shown to facilitate recombination of the viral genome with the cellular genome.

### 3.2. Prokaryotes

Early evidence for the presence of inverted repeats and cruciforms in genomes was obtained from studies across a range of bacteria, with a particular focus on *E. coli* [6]. Because bacterial DNA is often circular, it easily results in a negative supercoiled conformation [52], which can be an important factor in the formation of cruciforms. In the *E. coli* genome, short inverted repeats with arm lengths from 5 bp up to 20 bp are abundant in both coding and non-coding regions [19]. On average, there are nine inverted repeats per non-coding region, although a small proportion of regions contain the majority of the inverted repeats. The average arm length of the inverted repeats is approximately 6 bp, suggesting the sequences can form stable cruciforms. When comparing the genome with other proteobacteria, a significant number of identical inverted repeats are observed, providing evidence for evolutionary conservation [19]. Another study of genome sequences [53] performed similar analyses on 37 genomes of various prokaryotes, namely archaea, chlamydiales, firmicutes, proteobacteria, and others. For all bacteria, inverted repeats were found more frequently in non-coding regions. In almost all bacterial species examined, inverted repeats were found in genomes at a significantly higher frequency than the randomly generated sequences. Notably, only in two species, *Deinococcus radiodurans* and *Synechocystis* sp., were the observed number of inverted repeats statistically significantly lower than predicted by Markovian models of DNA sequences, although the reasons for the differences in these genomes are unclear. In archaea, the frequencies were higher than expected for five of eight species that were studied, but even in the five species that were higher, the difference was relatively small compared to that seen for bacteria. Mapping of the occurrence of inverted repeats in the *E. coli* genome [50] found that sequences with the potential to form cruciforms are enriched near stop codons and are part of terminators—and thus probably serve in the Rho-independent termination of transcription (Figure 3). Inverted repeats are also enriched within promoters, 5′-untranslated regions (UTRs), and in regions ~25–45 bp encompassing the start codon. It was also found that the small region ~5bp before the start codon has a statistically significant depletion of inverted repeats compared to 50 randomized genomes. Explanations for this observation could be that such a depletion prevents the formation of hairpin structures on the corresponding mRNA strands and also prevents disruption of the Shine Dalgarno sequence, both of which could negatively impact the initiation of translation.

For organisms that had complete genome sequences in 2020, about 36% of all bacteria and 75% of archaea have a prokaryotic immune system known as CRISPR/Cas [54]. CRISPR is an acronym for segments of clustered regularly interspaced short palindromic repeats, while Cas is the name of a group of proteins that associate with these regions. As the name implies, this system consists of sequences of inverted repeats, which are preceded by a leader sequence that is rich in adenine and thymine, and new spacers are integrated in its vicinity [55]. The nucleases Cas1 and Cas2 are the only Cas proteins that occur in all CRISPR/Cas systems, and both nucleases require a negatively supercoiled conformation to integrate new intervening sequences [56,57]. In vitro, the Cas1-Cas2 complex is able to integrate the new intervening sequence outside the CRISPR locus; however, the integration is non-random. In studying the specificity of integration of new intervening sequences, it was found that in the absence of the CRISPR locus, integration occurred preferentially in the vicinity of inverted repeats capable of forming cruciforms [56]. The CRISPR/Cas methodology is gaining widespread use across all organisms, but the potential impact of cruciforms on its implementation requires further analyses.

### 3.3. Eukaryotes

In eukaryotes, inverted repeats occur frequently in nuclear DNA and also in mitochondrial and plastid DNA, usually in even higher numbers than in nuclear DNA [20,21,58]. For example, in *Saccharomyces cerevisiae*, inverted repeats in mitochondrial DNA are 45-times more frequent than in its chromosomal DNA [17]. Correspondingly, inverted repeats have been demonstrated to impact evolution in mitochondria and in other genome contexts [59,60]. An overlay with annotated features revealed a statistically significant deficiency of inverted repeats in regions 20 bp downstream of the start codon [51]. In a similar way to examples already discussed for *E. coli* [50], inverted repeats in *S. cerevisiae* are enriched in the region ~ 30–60 bp downstream of the start codon and in close vicinity of positions corresponding to the ends of the mRNA (Figure 3) [51]. Whereas inverted repeats in *E. coli* are parts of intrinsic terminators and are GC-rich, inverted repeats in *S. cerevisiae* are parts of the polyA signal and are AT-rich. Therefore, inverted repeats in both organisms appear to play roles in transcription termination, although the sequences of the repeats are not preserved [50,51].

The effort to complete the sequence of the human genome is now successfully finished [61], with two chromosomes (8 and X) fully assembled already in 2021 [62,63]. Regions in chromosomes 8 and X that were uncharacterized in the current reference human genome assembly GRCh38 are now resolved and reveal a previous strong underestimation of the frequency of repeat tracts [64,65]. The difference of inverted repeat frequency between the two assemblies of chromosome 8 increases with the length of the inverted repeat, with up to twice as many for inverted repeats with an arm length of 30 bp [64]. When examining inverted repeats in promoters of the human genome [18], it was found that their frequency depends on the length of the repeat and its distance from the transcription start site. Shorter inverted repeats (6–11 bases for the size of the stem) are found primarily near the transcription start site, while longer repeats (14 bases and above for the size of the stem) are more frequent in regions that are at least 500 bp upstream from the transcription start site. In general, inverted repeats in the human genome are abundant upstream from the transcription start site, while downstream (in the direction of transcription), their presence is rarer (Figure 3). Some evidence suggests DNA is negatively supercoiled upstream of RNA polymerase [66], which will facilitate DNA strand separation and increase the likelihood that inverted repeats could form cruciforms [67]. The increased incidence of inverted repeats upstream of the transcription site would be consistent with these repeat sequences being involved in organizing and controlling promoter activities whether or not they form cruciforms [18,68]. It is also likely that the inverted repeats or potential cruciforms may impact differently on different transcription factors, as evidenced by promoters of genes involved in inflammatory, tumour, and developmental processes containing relatively high levels of inverted repeats, whereas promoters of metabolic-related genes contain lower levels of inverted repeats [18].

## 4. A Range of Proteins Interact with Cruciforms

Inverted repeats and cruciforms are targets for binding by many architectural and regulatory proteins. While many proteins have only weak sequence specificity, they are able to bind strongly to non-B-DNA structures, such as cruciforms [15]. Additionally, some proteins induce or stabilize cruciforms after binding to the nucleic acid. Cruciform binding proteins have been shown to have roles in chromatin remodelling, replication, and transcription regulation. Table 1 highlights the names and sources of proteins confirmed to interact with cruciforms, and details about the impact of some of these interactions have been discussed previously [15]. More recent findings in relation to the involvement of these interactions across the full range of cellular processes are described below.

Cruciform formation is enabled by DNA negative supercoiling, which is unevenly spread through genomes and is tightly regulated, mainly by topoisomerases (TOPs) [15]. In eukaryotes, TOP1 relaxes DNA supercoiling generated by transcription, replication, and chromatin remodelling through the introduction of a single-strand break, and it binds to Holliday junctions, whereas TOP2 changes the DNA topology and is capable of generating transient DNA double-strand breaks [99]. TOP2 has been shown to recognize and cleave cruciform structures [15]. TOP2 and a member of the HMG family, chromatin-stabilizing protein Hmo1, preserve negative supercoiling at gene boundaries and are suggested to instigate the formation of cruciforms, thus directing TOP1 and RNA polymerase II to coding regions [96].

Inverted repeats located in the promoter regions of genes are preferentially bound by many transcription factors (Table 1), such as PARP-1, BRCA1, ER, and p53 [15,70,90]. The tumour suppressor protein p53 is critical for protection against many human cancers. Most tumorigenic p53 mutations occur in its central domain, which binds to specific DNA sequences, referred to as response elements. Such response elements with a propensity to form cruciforms are favoured for binding by p53 both in vitro and in vivo [14,100]. The protein p73 is a member of the p53 family and has essential functions in several signaling pathways involved in development, differentiation, DNA damage responses, and cancer. Like its p53 homolog, p73 shows a preference for binding to its target sequence in cruciform structures [89]. Yeast-based assays revealed that p73-mediated transactivation correlated with the relative propensity of a response element to form a cruciform [89].

Another protein showing a preference for binding to DNA cruciforms is interferon-inducible protein 16 (IFI16), a sensor of foreign DNA in human cells. Upon DNA recognition, the protein oligomerizes, forms a filament, and triggers an innate immune response [101]. Besides its role in the immune response, IFI16 represses the transcription of viral genes [102]. IFI16 showed a preference for binding to negatively supercoiled plasmid over linear DNA in vitro, stabilizing local DNA structures such as cruciforms and quadruplexes [83]. Importantly, the binding pattern varies dependent on secondary structures in the DNA: with linear DNA, the protein interacts cooperatively, leading to non-specific filamentous aggregates of a higher molecular weight being formed, but in the presence of cruciforms, the protein binds to DNA selectively, forming more compact globular complexes [83,84]. The functional role of the different binding patterns remains unclear, but provides a possible explanation for the distinct roles of IFI16 in antiviral defence.

Cruciforms have also been demonstrated to influence various aspects of DNA replication. A range of studies confirmed cruciform formation in the origins of replication in bacteria, yeast, and mammalian cells [15,103]. Furthermore, several proteins involved in replication bind to cruciform structures, such as S16, MLL, WRN, and 14-3-3 (Table 1). Replication initiator protein C (RepC), which is encoded by the pT181 plasmid of *Staphylococcus aureus*, binds to a specific DNA sequence, which is able to form a cruciform and creates a nick that allows replication to begin [104]. It is proposed that cruciforms are formed passively due to the natural supercoiling of DNA, but their formation is necessary for RepC cleavage of DNA [92]. Rap1-interacting factor 1 (Rif1) is a mammalian protein involved in regulating the timing of DNA replication, mediating the repair of double-stranded DNA breaks, and replication fork restart [93]. The C-terminal region CII of RIF1 is critical for replication fork protection, and recent structural analyses identified that it preferentially binds cruciform structures [93,94]. Rif1 accumulates on stalled replication forks and possibly protects reversed forks, which could involve cruciform structures in vivo.

Cruciforms also influence other aspects of replication. Cruciforms formed ahead of a replication fork could stop their movement, which would temporarily stop replication. Such problems can be resolved by the formation of reversed replication forks at the four-way junctions, followed by homologous recombination and branch migration in order to restart replication [105]. Since cruciforms share structural similarity with Holliday junctions, cruciform-binding proteins are likely to be involved in these (or related) processes. For example, AT-rich cruciform cleavage is mediated by the Holliday junction resolvase GEN-1 in human cells [79,106], with GEN1 splitting the cruciform diagonally, creating two hairpins healed by DNA ligases [79]. The tips of these hairpins are then cleaved by Artemis proteins and joined by non-homologous end joining. The resulting heteroduplexes are repaired by proteins associated with mismatch repair (MMR), for which the template would normally be selected according to the strand where the nick is not ligated. Since, however, both strands are fully ligated, the template is chosen randomly and may result in translocation between two palindromic AT-rich repeats at different chromosomal locations that do not share a complete sequence homology. The involvement of other resolvases in this type of process, such as Mus81 in human cells, was rejected [79]. However, in *S. cerevisiae*, Mus81-Mms4 was able to process recombination intermediates that arose during the repair of stalled replication forks and double-stranded breaks after being stimulated by Crp1, a protein that specifically binds to DNA four-way junctions [72,107].

Notably, long inverted repeats with an arm length of more than 150–200 nucleotides and with a spacer between the repeats being shorter than 50–60 nucleotides are almost impossible to clone into *E. coli*, mainly due to the action of SbcCD endonuclease/exonuclease, which can cleave hairpin structures, leading to DNA double-strand breaks [95,108]. It was confirmed that such long inverted repeats are converted to cruciform DNA before they encounter the replication fork, creating SbcCD-sensitive hairpin structures on both leading and lagging strands that transiently impede replication fork movement [109].

Another example of a protein able to bind to cruciforms is DNA-binding protein from starved cells (Dps), which is produced in stationary-phase *E. coli* cells on a large scale, reaching 85,000–180,000 molecules per cell. The main role of Dps is to protect cells from oxidative stress, UV- and γ-radiation, and metal ion toxicity, which it does via its ferroxidase activity [75]. Dps also regulates transcription by competing for binding sites with other transcription factors [76]. Dps protein binding to DNA does not depend on sequence, but a non-random distribution of Dps binding sites was observed with significant correlation with inverted repeats, suggesting the protein may interact with specific structures in DNA [76,77].

## 5. Inverted Repeats and Cruciforms as Potential Therapeutics in Human Disease

**Figure 4:**
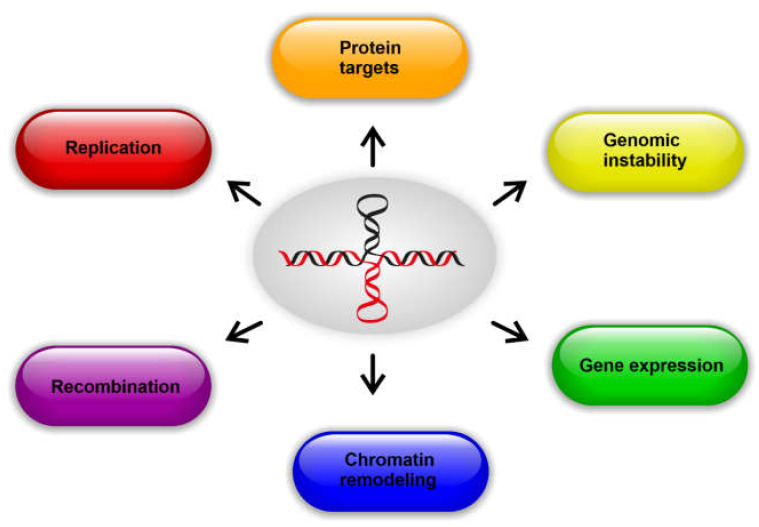
Cellular processes influenced by cruciform structures.

Evidence presented so far clearly demonstrates that cruciforms can form within DNA molecules in cells and that proteins bind to them, but the physiological significance of these observations remains unclear, particularly for human cells. However, a recent analysis of 1000 human genomes estimated that the probability of occurrence of pathology-associated single-nucleotide polymorphism variants is 14-times higher in inverted repeats than in other genome sites [110], and their role has been shown in germline mutagenesis with implications for evolution and genetic diseases [111]. Single-nucleotide polymorphism variants in inverted repeats have been linked with many human neuronal disorders, mental retardation, and various cancers. Moreover, when amplified genomic regions are determined for various cancer types [112], short palindromes are observed to facilitate these processes and lead to cancer progression [113]. Due to the presence of inverted repeats in multiple parts of genomes that are associated with regulatory functions, cruciforms are likely to be involved in several basic biological processes with physiological and pathological importance (Figure 4).

A range of local DNA structures are suggested as good therapeutic targets for human disease [9,114]. Considering that cruciforms formed by inverted repeats are hotspots of DNA breakpoints and for mutations with various pathologies [27,48], the detailed knowledge presented within this review provides an important background for their use as therapeutic targets. Incomplete assemblies of genomes present significant problems in that sequences with good potential to form local DNA structures are often not characterized properly, and until recently, many repeat tracts have not been identified because sequencing technologies have not been able to cope with them [64]. Fortunately, contemporary sequencing technologies allow the complete assembly of even very complex genomes, including the human genome [63]. As described above, recent data of complete human chromosomes identified inverted repeats in the human genome that had previously not been seen [64]. The improved understanding of the widespread nature of these regulatory sequences will make it possible to judge more accurately whether their targeting is feasible for specific human diseases.

The range of structures that can be adopted within DNA have important impacts on genome integrity and genome plasticity. Thus, it is not surprising that cruciforms (and four-way junctions) play critical roles in the maintenance of genomic stability, with a concomitant impact on essential cellular processes [15,115]. For example, this is observed directly through their identification as hotspots of genomic rearrangements [115]. Molecular mechanisms have been inferred for how these types of structures mediate such rearrangements in the human genome [116], such as by Holliday junction resolvases mediating chromosomal translocations, as discussed above. Inverted repeats are frequently found at fragile sites in the genome that are prone to chromosome breakage, as shown for the fragile site FRA16D, where a variable-length AT repeat forms a cruciform that stalls replication [117]. The relative position and size of inverted repeats is also important in relation to their effects on genome stability. These parameters impact translocation frequency, with an inverted repeat arm size of up to 100 bp correlating with translocation breakpoints in human cancer genomes [97]. The involvement of structure-specific nucleases on the fragility of inverted repeats also depends on the distance between them and their transcriptional status [87]. The association of several human diseases with mutations of DNA helicases has also suggested possible roles for cruciforms in the diseases [118]. Although cruciforms may be important for basic biological processes, if they are not resolved by helicases, their presence could lead to transcription stop or delay and to chromosome breakage during replication. Dysfunction of these helicases can lead to various diseases, for example Werner’s syndrome, which is associated with mutations in the WRN helicase [119]. Inverted repeats also play a key role in the transposition and reorganization of transposable elements as demonstrated in several disease models, for example in Williams–Beuren syndrome, where insertions and deletions are associated with genomic regions that have an abundant number of inverted repeats [120].

Cruciforms are already used for various applications in medicine. For example, a cruciform DNA nanostructure is used for targeted delivery of doxorubicin to cancer cells [121] and was used to treat colon cancer [122]. It has also been demonstrated that cruciforms in gene promoters impact transcription upon oxidative modification of 2’-Deoxyguanosine [123]. The association of cruciforms with the regulation of transcription [90], as discussed above, opens other therapeutic windows where the specific levels of gene expression are influenced by the their presence and stability in promoter regions. An important tool allowing such approaches is the monoclonal antibody with specificity to the cruciform structure, although up to now, this has only been used for research purposes [28,69,124]. Currently, there are no small molecules that specifically recognize cruciforms, but it is likely that compounds will soon be designed that impact cruciform–protein interactions.

## 6. Conclusions

DNA molecules that contain inverted repeat sequences are able to adopt fully base-paired “linear” conformations and cruciforms that contain several unpaired regions. The structures of cruciforms (and four-way junctions) have been best characterized in vitro, including in complexes with proteins from prokaryotes and eukaryotes that bind to hairpins and four-way junctions. The structure of the cruciform influences the thermodynamic stability of the DNA, and paired regions of at least 6 bp are usually required to offset the energetically unfavourable folding of the junction and loop regions. In recent years, significant advances have been made in identifying high-resolution analyses of unusual DNA structures, either as the nucleic acid alone or in combination with proteins. A range of structural studies has demonstrated that cruciforms and four-way junctions adopt different structures depending on the ionic environment and other factors, including whether or not proteins are interacting with them. High-resolution structures that are currently available for four-way DNA junctions are usually for sequences that are not inverted repeats, but it is expected that structures formed by inverted repeats will have similar flexibility, although this still has to be verified by high-resolution structures. It will be useful to confirm at high resolution whether proteins bind to the junction, stem, or loops, or whether this is protein-dependent.

Detailed studies of many organisms have identified that inverted repeats are widespread in natural genomes. Indeed, in most cases, they are found at higher levels than expected if these were present at just random frequencies. This suggests that these types of sequences and/or their structures have functions in cells. In most eukaryotes, inverted repeats occur in higher amounts near promoters and transcriptional terminators, whereas in prokaryotes, they occur more frequently close to terminators. Both observations suggest these sequences and/or their cruciform structures have roles in regulating transcription. A similar increase in the amount of inverted repeats occurs near the origins of replication in eukaryotes, suggesting that the proteins involved in the initiation of replication may bind to these sequences and/or the structures within them.

The presence of inverted repeats can have negative effects on genome stability, and they have been shown to promote mutations and are, thus, an important driver of evolution. When examined in relation to human diseases, such as a range of cancers, genetic rearrangements are often abundant and complex, meaning it can be difficult to unravel the events that start and then lead to a certain genotype. Clearly, amplifications of inverted repeats have important impacts on the mechanisms involved in carcinogenesis, but their exact roles in diseases remain unclear; those that exist in the human genome could have a much greater role in initiating recombination events than is currently appreciated.

Although inverted repeats have been the subject of many studies over the last 50 years, their distribution has recently become an increased focus of research due to developments in sequencing and computer software. It is now clear that inverted repeats are conserved and not randomly distributed in genomes, suggesting that they play important roles in nucleic acid metabolism. In the future, advances with in vitro and in vivo methods will allow experimental examination of the predictions from bioinformatics analyses, facilitating thorough investigations into the effects of cruciforms on cellular processes, providing a deeper understanding of the resulting effects on human disease.

## Figures and Tables

**Table 1:** Proteins involved in interactions with cruciform structures. TF = transcription factor, chromatin AP = chromatin-associated protein. Adapted from [15]. * If no reference is listed for an entry, see [15] for further details.

Protein	Source	Function	Reference *
**14-3-3**	Eukaryotes	Replication, DNA repair, TF	[69]
**A22**	Coccinia virus	Junction-resolving enzyme	
**AF10**	*H. sapiens*	TF	
**Bmh1, homolog of 14-3-3**	*S. cerevisiae*	Replication, DNA repair, TF	
**BRCA1**	Mammals	Chromatin AP, DNA repair, TF	[70]
**Cas1, Cas2**	Archaea, Bacteria	Endonuclease, defence response to virus	[56,57]
**Cce1**	Yeast	Junction-resolving enzyme	[71]
**Crp-1**	*S. cerevisiae*	DNA repair	[72]
**DEK**	Mammals	Chromatin AP, replication, DNA repair	[73,74]
**DNA-PK**	Eukaryotes	DNA repair	
**Dps**	*E. coli*	DNA repair, stress response	[75,76,77]
**Endonuclease I**	Phage T7	Junction-resolving enzyme	[78]
**Endonuclease VII**	Phage T4	Junction-resolving enzyme	
**Estrogen receptor**	Mammals	TF	
**GEN1**	Vertebrates	Junction-resolving enzyme	[79]
**GF14, homolog of 14-3-3**	Plants	Replication, stress response	
**Helicases**	all	Replication	[80,81]
**Hjc, Hje**	Archaea	Junction-resolving enzymes	
**HMG protein family**	all	Chromatin AP, DNA repair, TF	
**Hop1**	*S. cerevisiae*	DNA Repair	
**HU**	*E. coli*	Replication	[82]
**IFI16**	*H. sapiens*	Viral DNA recognition	[83,84]
**Integrases**	all	Junction-resolving enzyme	
**MLH1-MLH3**	Vertebrates	Junction-resolving enzyme	[85]
**MLL (leukaemia)**	*H. sapiens*	Replication	
**MSH2**	Mammals	Junction-resolving enzyme	[86]
**Mus81-Eme1**	Eukaryotes	Junction-resolving enzyme	
**Mus81-Mms4**	*S. cerevisiae*	Junction-resolving enzyme	[72,87]
**MutH**	Eukaryotes	Junction-resolving enzyme	
**p53**	*H. sapiens* and others	DNA repair, TF	[88]
**p73**	*H. sapiens* and others	DNA repair, TF	[89]
**PARP-1**	*H. sapiens* and others	DNA repair, TF	[90]
**Rad51**	Eukaryotes	Chromatin AP	[91]
**Rad52-Rad59**	Eukaryotes	Chromatin AP	[91]
**Rad54**	Eukaryotes	Chromatin AP	[91]
**RecU**	G+ bacteria	Junction-resolving enzyme	
**RepC**	Bacteria	Replication	[92]
**Rif1**	Mammals	DNA repair, TF	[93,94]
**Rmi-1**	Yeast	DNA repair, TF	
**RusA**	*E. coli*	Junction-resolving enzyme	
**RuvC**	*E. coli*	Junction-resolving enzyme	
**S16**	*E. coli*	Replication	
**SbcCD**	*E. coli*	Junction-resolving enzyme	[95]
**Smc**	*S. cerevisiae*	DNA repair, TF	
**Topoisomerase I**	Eukaryotes	Chromatin AP	
**Topoisomerase II**	Eukaryotes	Chromatin AP	[96]
**TRF2**	*H. sapiens*	Junction-resolving enzyme	
**Vlf-1**	Baculoviruses	Replication	
**WRN(Werner syndrome)**	*H. sapiens*	Replication	
**XPF, XPG protein families**	Eukaryotes	Junction-resolving enzyme	[97]
**Ydc2**	*S. pombe*	Junction-resolving enzyme	
**Yen1, homolog of GEN1**	*S. cerevisiae*	Junction-resolving enzyme	[98]
